# Successful Endoscopic Retrograde Appendicitis Therapy Following a Recent ST-Elevation Myocardial Infarction

**DOI:** 10.14309/crj.0000000000001324

**Published:** 2024-03-29

**Authors:** Zoilo K. Suarez, Jalal Samhoun, Joshua Drourr, Talwinder Nagi, Muhammad A. Haider, Charles Vallejo, Zahra Touqir, David Forcione

**Affiliations:** 1 Internal Medicine Department, Florida Atlantic University Charles E. Schmidt College of Medicine, Boca Raton, FL

**Keywords:** ERAT, DAPT, STEMI, endoscopic retrograde appendicitis therapy, appendicitis

## Abstract

Acute appendicitis is one of the most common abdominal surgical emergencies. A laparoscopic or open appendectomy has traditionally been the gold standard. Antibiotic therapy has recently been found to be noninferior. The treatment of acute uncomplicated appendicitis remains a challenge, especially in the presence of an appendicolith. We present a case of a 59-year-old man with recent ST-elevation myocardial infarction who underwent successful endoscopic retrograde appendicitis therapy.

## INTRODUCTION

Acute appendicitis is one of the most common abdominal surgical emergencies. Currently, the management of acute uncomplicated appendicitis may be done conservatively or surgically. The presence of an appendicolith has been associated with a higher risk of complications. A novel endoscopic approach has been proposed as an effective alternative. We present a case of a 59-year-old man with recent ST-elevation myocardial infarction who underwent successful endoscopic retrograde appendicitis therapy (ERAT).

## CASE REPORT

**Figure 1.**
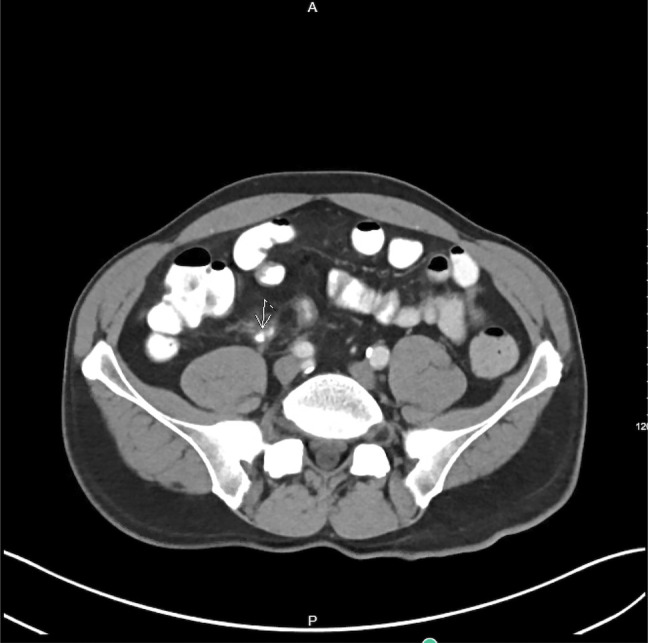
Computed tomography scan of the abdomen and pelvis with intravenous contrast showing a fecalith at the appendiceal orifice with reactive terminal ileum wall thickening.

A 59-year-old man with a history of coronary artery disease who underwent a percutaneous coronary intervention 2 weeks earlier presented to the emergency department for evaluation of abdominal pain in the right lower quadrant. He was found to have a white blood cell count of 14.1 × 10^9^/L, and his comprehensive metabolic panel was unremarkable. An abdominal computed tomography scan showed inflammatory changes surrounding the appendiceal tip and wall. The Alvarado score was 8 points. He was given intravenous fluids and started on piperacillin-tazobactam, and general surgery and cardiology were consulted. Given the patient required adherence to dual-antiplatelet therapy with aspirin and ticagrelor, he was considered at high risk of bleeding, for which conservative management was pursued. Subsequently, blood cultures from admission had no growth, and after 6 days of antibiotic therapy, a repeated abdominal computed tomography scan showed a fecalith at the appendiceal orifice (Figure 1).

**Figure 2.**
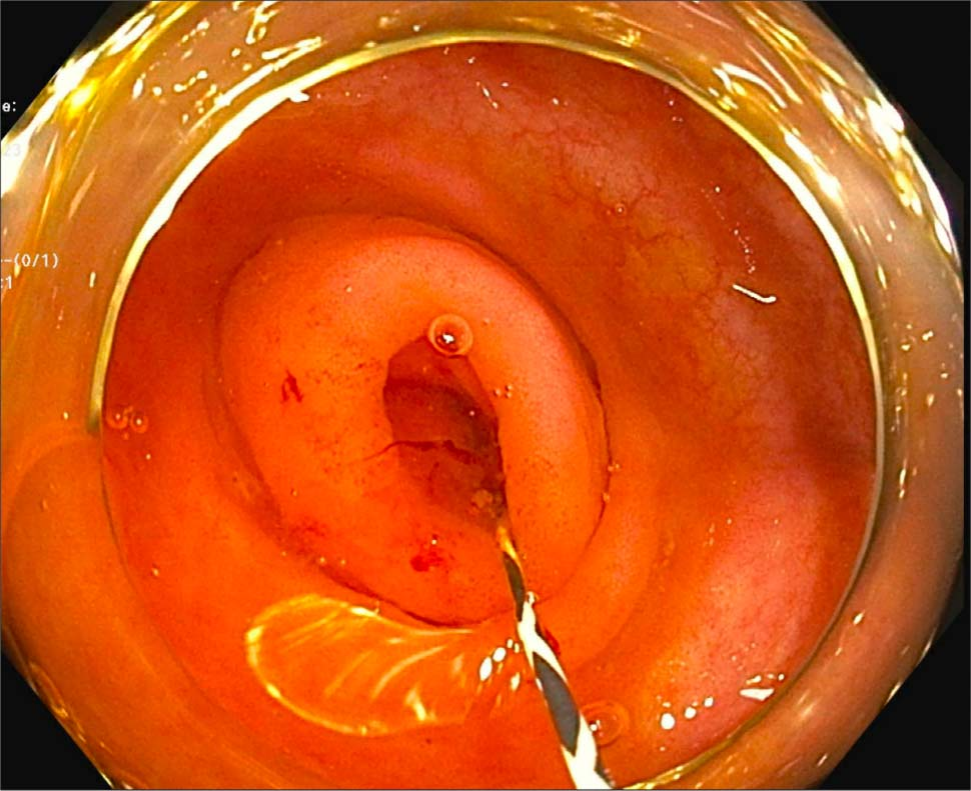
Cap-fitted Olympus adult colonoscope visualizing the cecum with a 0.025-inch guidewire into the appendiceal lumen.

**Figure 3.**
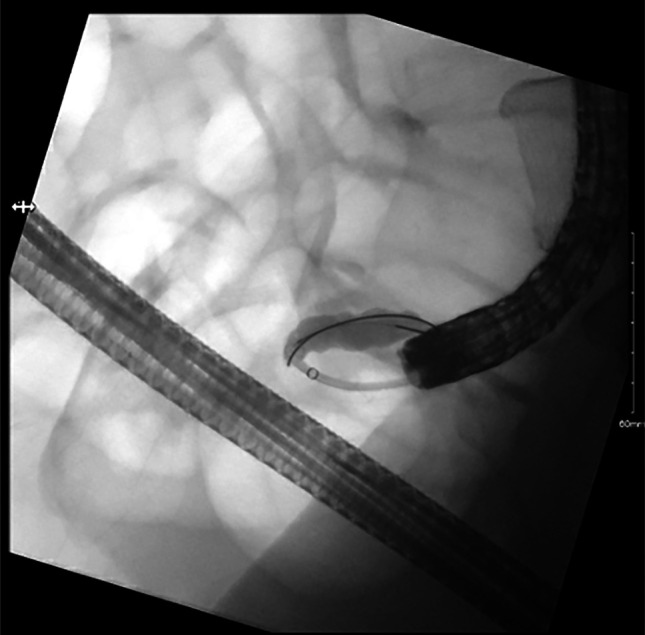
Endoscopic retrograde appendicography fluoroscopy with minor filling defects in the appendix lumen.

**Figure 4.**
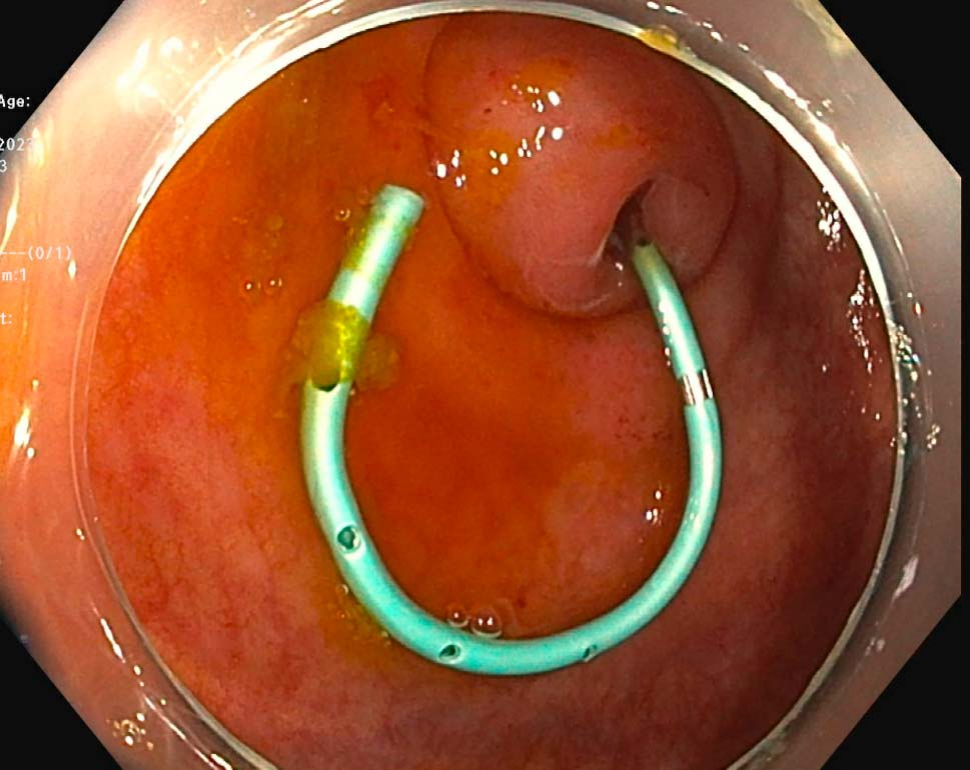
A 5 French 8 cm single pigtail pancreatic stent into the appendiceal lumen.

Interventional endoscopy was consulted, and after multidisciplinary conversations, the decision was made to pursue source control with ERAT. The patient received monitored anesthesia care with propofol. A cap-fitted Olympus adult colonoscope was advanced through the anal canal into the rectum under direct vision. Subsequently, it was advanced to the cecum. The appendiceal orifice was noted to be edematous and mildly hyperemic with no purulence. A 0.025-inch guidewire (manufactured by Boston Scientific) was advanced into the appendiceal lumen under fluoroscopic guidance (Figures 2 and 4). A 7 French Soehendra catheter was passed, followed by irrigation of the appendiceal lumen with a combination of gentamicin and metronidazole solution. Following irrigation, soft debris was expelled with lavage and a 5 French 8-cm single-pigtail pancreatic stent was deployed into the lumen to maintain drainage (Figure 3). The scope was withdrawn without evidence of additional lesions and complications. Bowel preparation quality was excellent, consistent with a total Boston Bowel Preparation Score of 9.

Ultimately, the patient was discharged 2 days later on a 1-week course of amoxicillin-clavulanate and recommended to follow up with interventional endoscopy, cardiology, and an infectious disease specialist. Upon follow-up 6 months after ERAT, the patient has remained asymptomatic and has not required any further intervention (Figure 4).

## DISCUSSION

Acute uncomplicated appendicitis is a common abdominal emergency that requires prompt medical management. A laparoscopic or open appendectomy has traditionally been considered the gold standard.^1^ Antibiotic therapy was found to be noninferior to appendectomy by the Comparison of Outcomes of Antibiotic Drugs and Appendectomy trial; however, the presence of an appendicolith was linked to a higher risk of complications due to the inability to achieve source control.^2^

ERAT was described in 2012 in China as a minimally invasive therapeutic approach for acute uncomplicated appendicitis.^3^ In our case, ERAT successfully achieved source control in a patient who was deemed at high risk of bleeding in the setting of dual-antiplatelet therapy with aspirin and ticagrelor.^4^ ERAT provides the benefit of removing the obstruction, providing control of the culprit as well as symptomatic relief while minimizing the risk of bleeding and avoiding the risk of perioperative coronary stent thrombosis and the need for bridging antiplatelets.^5^ In addition, it has been associated with shorter procedural duration and length of hospital stay.^6^ It has also been proposed to be a sustainable and cost-effective alternative.^7^ However, the risk of recurrence of acute appendicitis remains a challenge and has been estimated to be between 6.01% and 7.9%.^8–10^ However, it could be decreased by appendiceal stent placement.^9^ Further studies are needed to establish indications for appendiceal stent placement and identify factors associated with recurrence.^11^

In summary, the implementation of an endoscopic organ-preserving approach led to the effective management of acute uncomplicated appendicitis. Evaluation by a multidisciplinary team including an advanced endoscopist, abdominal surgeon, and cardiologist is the cornerstone for comprehensive care.

## DISCLOSURES

Author contributions: ZK Suarez, J. Drourr, MA Haider and C. Vallejo: participated in patient care. ZK Suarez, J. Samhoun and T. Nagi: collaborated in the data collection and literature search. ZK Suarez: collaborated in manuscript elaboration. Z. Touqir and D. Forcione: collaborated in the critical revision of the manuscript for important intellectual content. ZK Suarez is the article guarantor.

Financial disclosure: None to report.

Previous presentation: An abstract of the case report was presented at the 2023 Florida Gastroenterologic Society Annual Meeting. September 8th–10th, 2023 in Orlando, Florida

Informed consent was obtained for this case report.
